# 5.0T multi-parametric diffusion MRI histogram analysis for prostate cancer pathology grading and adverse pathological factors

**DOI:** 10.3389/fonc.2026.1957407

**Published:** 2026-09-29

**Authors:** Sen Xing, Ying Tan, Xinghua Liu, Yong Luo, Yun Wen, Huan Ma, Shaoxin Xiang, Yixin Emu, Yao Chen, Wenbing Zeng

**Affiliations:** 1 Department of Radiology, Chongqing University Three Gorges Hospital, Chongqing, China; 2 Department of Ultrasound, Chongqing University Three Gorges Hospital, Chongqing, China; 3 MR Research Collaboration, Shanghai United Imaging Healthcare Co., Ltd., Shanghai, China

**Keywords:** 5.0-T, diffusion kurtosis imaging, diffusion weighted imaging, histogram analysis, intravoxel incoherent motion, prostate cancer, ultra-high-field magnetic resonance

## Abstract

**Objective:**

To evaluate 5.0-T ultra-high-field multi-parametric diffusion MRI (mp-dMRI) combined with histogram analysis for assessing the pathological grade, stage, and adverse pathological factors in prostate cancer (PCa).

**Methods:**

This retrospective study included 138 patients with histopathologically confirmed PCa. All patients underwent preoperative 5.0-T MRI, including diffusion-weighted imaging (DWI), diffusion kurtosis imaging (DKI), and intravoxel incoherent motion (IVIM) sequences. Histogram parameters were extracted from parametric maps of the apparent diffusion coefficient (ADC), mean diffusivity (MD), mean kurtosis (MK), true diffusion coefficient (D), perfusion fraction (f), etc. using 3D Slicer software. Between-group comparisons were performed using the Mann-Whitney U test or the independent-samples t-test, as appropriate. Spearman’s rank correlation analysis was used to evaluate the relationships between histogram parameters and both the ISUP grade group (ISUP GG) and pathological T stage (pT). Logistic regression was used for variable selection and model construction. Diagnostic performance was assessed using receiver operating characteristic (ROC) curve analysis.

**Results:**

Among the 138 patients, most histogram parameters of ADC, MD, MK, D, and f differed significantly between clinically significant PCa (csPCa) and clinically insignificant PCa (ciPCa). For distinguishing csPCa from ciPCa, D_90th percentile achieved the highest AUC among single parameters (0.756). Univariate and multivariate logistic regression analyses identified ADC_mean, MD_Minimum, D_90th percentile, and f_Maximum as independent predictors across the DWI, DKI, and IVIM sequences. The combined model comprising these four parameters achieved an AUC of 0.809. After internal validation using bootstrap resampling (1,000 repetitions), the resulting AUC was 0.808, indicating good model robustness. Further correlation analysis showed that multiple histogram parameters of ADC, MD, MK, D, and f also demonstrated mild to moderate correlations with ISUP GG and pT stage. D_RMS and MD_skewness exhibited the strongest correlations with ISUP GG and pT stage, respectively (*r* = -0.458 and *r* = 0.443, both *P* < 0.001). Finally, among the 60 surgical patients, multiple histogram features of ADC, MD, MK, D, and f differed significantly between patients with and without EPE, SVI, PSM, and PNI.

**Conclusion:**

Quantitative histogram parameters derived from 5.0-T ultra-high-field mp-dMRI show promise for the non-invasive assessment of tumor aggressiveness and adverse prognostic factors in PCa.

## 1 Introduction

According to the latest data from the World Health Organization, prostate cancer (PCa) has become the second most common cancer among men worldwide, with over 1.46 million new cases and 370,000 deaths annually (1). Of particular concern is the rising incidence of clinically significant disease (csPCa), which not only increases the risks of metastasis, recurrence, and adverse outcomes but also complicates clinical management and treatment strategies (2). The International Society of Urological Pathology Grade Group (ISUP GG) (ISUP GG 1-5, corresponding to Gleason scores (GS) ≤ 6, 3 + 4, 4 + 3, 8, and 9-10) offers advantages for identifying csPCa, assessing tumor heterogeneity, and facilitating multidisciplinary communication (3). Specifically, ISUP GG 1 denotes clinically insignificant prostate cancer (ciPCa), which is typically indolent and managed with active surveillance, whereas ISUP GG 2–5 denotes csPCa requiring active intervention (4). Beyond ISUP GG and pathological T (pT) stage, adverse pathological factors such as extraprostatic extension (EPE), seminal vesicle invasion (SVI), positive surgical margins (PSM), and perineural invasion (PNI) are closely associated with PCa prognosis (5–8). Additionally, patients harboring these adverse pathological factors may require adjuvant therapies (e.g., radiotherapy or antiandrogen therapy) following definitive surgical treatment (9). Therefore, non-invasive and accurate preoperative risk assessment is critical for clinical decision-making and improving long-term prognosis.

The Prostate Imaging Reporting and Data System (PI-RADS) based on magnetic resonance imaging (MRI) is currently a core tool for PCa diagnosis and assessment (10). However, the absence of quantitative imaging parameters for assessing tumor biological behavior renders PCa diagnosis and prognostic assessment dependent on subjective morphological interpretation, thereby hindering accurate evaluation of tumor aggressiveness and adverse prognostic factors. As a texture-based statistical method, histogram analysis reflects overall lesion microstructural characteristics and, more importantly, enables objective and comprehensive quantification of intratumoral heterogeneity (11). This approach has shown increasing promise for PCa subtyping and prognostic assessment (12, 13). Meanwhile, growing evidence supports the value of multi-parametric diffusion MRI (mp-dMRI)—encompassing diffusion-weighted imaging (DWI), diffusion kurtosis imaging (DKI), and intravoxel incoherent motion (IVIM)—for evaluating PCa microstructure and pathological features (14, 15). Integrating mp-dMRI with histogram analysis thus enables comprehensive characterization of tumor heterogeneity and improved prognostic assessment (16). Moreover, this approach is non-invasive and contrast-agent-free, offering particular advantages for patients with renal impairment or gadolinium contraindications (17).

The combination of mp-dMRI parameters and histogram analysis has been applied for PCa evaluation at 1.5-T and 3.0-T (18, 19). Although this approach has been used for PCa detection and grading, its utility for preoperative assessment of adverse prognostic factors remains constrained by field strength and spatial resolution (20). Recently, 5.0-T ultra-high-field MRI has emerged as a clinically viable whole-body imaging platform. Studies have shown that (21), compared with 1.5-T and 3.0-T systems, 5.0-T MRI offers superior signal-to-noise ratio and spatial resolution, enabling improved visualization of anatomical details and detection of subtle lesions. Meanwhile, recent studies have further demonstrated the value of 5.0-T MRI in brain (22), heart (23), and kidney (24) imaging. However, its application in PCa—specifically for preoperative assessment of pathological grade, stage, and adverse prognostic factors—remains largely unexplored.

This study aimed to evaluate ISUP GG and pT stage, and to preliminarily explore adverse pathological factors in PCa using 5.0-T ultra-high-field MRI combined with multi-parametric diffusion-weighted imaging (DWI, DKI, and IVIM) and histogram analysis.

## 2 Materials and methods

### 2.1 Study population

This single-center retrospective study combined prospective data acquisition with retrospective analysis. We included patients with histopathologically confirmed PCa who underwent 5.0-T prostate MRI at Chongqing University Three Gorges Hospital between July 2024 and December 2025. Of the 160 patients initially screened, 22 were excluded because of incomplete imaging or clinical data, insufficient image quality for diagnostic evaluation, or a target lesion diameter < 5 mm. Ultimately, 138 patients were included in the final analysis: 108 with csPCa and 30 with ciPCa. Of these, 60 patients underwent radical prostatectomy (RP). All pathological diagnoses were established based on ultrasound-guided biopsy or radical prostatectomy specimens. When both types of specimens were available, radical prostatectomy findings served as the reference standard. To assess the agreement between biopsy-based and RP-based grading, a subgroup validation analysis was performed in patients with paired preoperative biopsy and RP specimens.

Inclusion criteria: 1) histopathological confirmation by ultrasound-guided biopsy or radical prostatectomy within one month after MRI; 2) no history of other malignancies; 3) no prior prostate intervention (e.g., surgery, radiotherapy, or endocrine therapy) before MRI.

Exclusion criteria: 1) incomplete imaging or clinical data; 2) insufficient MRI image quality for diagnostic evaluation; 3) target lesion diameter < 5 mm. This study was conducted in strict accordance with the Declaration of Helsinki and was approved by the Institutional Review Board and Ethics Committee of Chongqing University Three Gorges Hospital (Approval No. KS-2024072). Because of the retrospective design, the requirement for informed consent was waived per institutional guidelines. The patient screening process is depicted in **Figure 1**.

**Figure 1:**
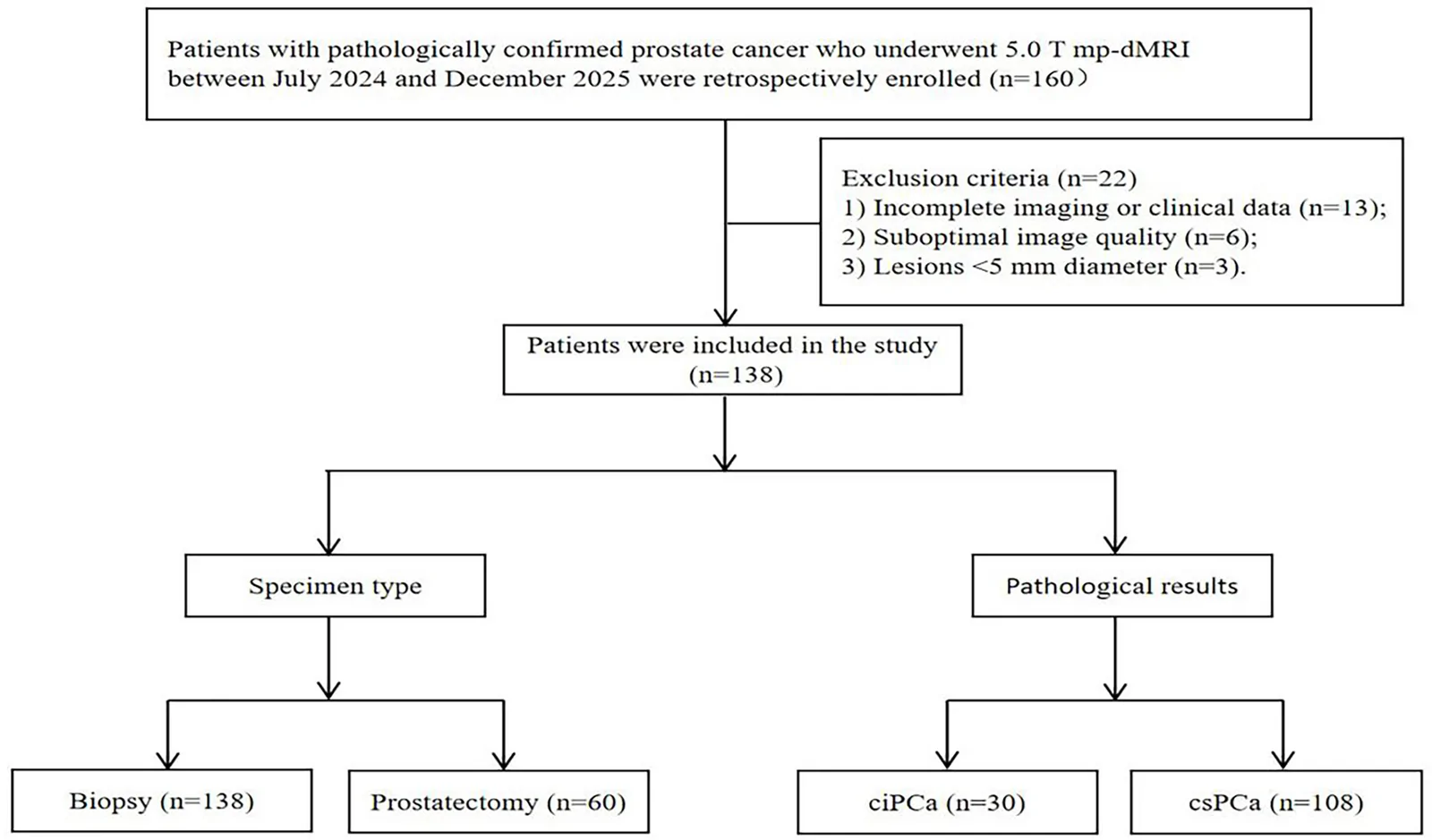
Patient selection flowchart.

### 2.2 Imaging protocol

All examinations were performed on a 5.0-T scanner (uMR Jupiter, United Imaging Healthcare, Shanghai, China) with a combination of a 24-channel flexible body array coil and a 24-channel integrated spine coil. Detailed acquisition parameters are summarized in **Table 1**.

**Table 1:** MRI scanning sequence parameters.

Scanning parameters	T1WI	T2WI	DWI	DKI	IVIM
Sequence	FSE	FSE	EPI	EPI	EPI
TR (ms)	624	8451	2483	3194	4507
TE (ms)	10.0	147.9	55.0	55.0	68.0
Slice thickness (mm)	3.0	3.0	3.0	3.0	4.0
Spacing (mm)	0.3	0.3	0.3	0.3	0.4
Fov (mm^2^)	220×220	220 × 220	220 × 220	250 × 220	260 × 260
Matrix	320×320	320×320	144 × 144	144 ×144	160 × 160
b value (mm^2^/s)	–	–	0, 50, 800, 1500	0, 50, 800, 1000, 1500, 2000	0, 10, 20, 30, 50, 100, 200, 400, 800, 1000, 1500
NEX	2	2	1, 1, 3, 10, 12	1, 1, 3, 10, 12	1, 1, 1, 1, 1, 2, 2, 3, 4, 4, 8
Scanning time (min:s)	1:53	2:08	2:32	4:28	6:30

T1WI, T1-weighted imaging; T2WI, T2-weighted imaging; FSE, fast spin-echo; EPI, single-shot echo planar imaging; TR, repetition time; TE, echo time; FOV, field of view; NEX, Number of Excitations.

### 2.3 Image processing and analysis

The original DWI, DKI, and IVIM data were processed on the 5.0-T uMR Jupiter MRI system to generate parametric maps of the apparent diffusion coefficient (ADC), mean diffusivity (MD), mean kurtosis (MK), true diffusion coefficient (D), pseudo-diffusion coefficient (D*), and perfusion fraction (f), and were exported in DICOM format. Blinded to pathological results, two genitourinary radiologists (S.X. and X.L., with 12 and 20 years of experience, respectively) independently performed three-dimensional image analysis and ROI delineation using 3D Slicer (version 5.4.0). ROIs were manually delineated layer-by-layer along the solid tumor boundaries, with reference to T1WI and T2WI images to exclude non-tumor regions (e.g., necrosis, hemorrhage, calcification, and periurethral vessels). For multifocal PCa, the index lesion (defined as the largest tumor focus) was selected for analysis. Lesions with a diameter ≥ 5 mm were included to cover the tumor burden, whereas those < 5 mm were excluded. For parameters derived from the same scan sequence (e.g., D, f, and D* from IVIM), the ROI was transferred to the corresponding parametric maps, with spatial accuracy confirmed by visual inspection. For parameters from different sequences (e.g., IVIM vs. DKI), the ROI was redrawn. **Figures 2** and **3** show representative images and delineations of ciPCa and csPCa, respectively. Image preprocessing and radiomics feature extraction were performed using the SlicerRadiomics extension module. Images were resampled to an isotropic voxel size of 1 × 1 × 1 mm³ using B-spline interpolation (sitkBSpline). For ROI masks, nearest-neighbor interpolation was used to preserve label integrity, and gray-level discretization was performed with a fixed bin width of 25. Radiomics features were extracted exclusively from the first-order histogram category.meter Extracted features included mean, median, root mean square (RMS), 10th and 90th percentiles, minimum, maximum, skewness, kurtosis, range, uniformity, etc. Based on their statistical properties, the extracted histogram features were classified into two categories—central tendency and dispersion parameters—to characterize voxel intensity distribution. This classification aligns with standard radiomics frameworks and enables quantitative assessment of intratumoral heterogeneity from complementary perspectives. Central tendency parameters (mean, median, and RMS) reflect the average voxel intensity within the tumor. Dispersion parameters (percentiles, skewness, kurtosis, range, and uniformity) quantify the degree of variation around the central value and directly reflect tissue heterogeneity (25). The intraclass correlation coefficient (ICC) was used to assess inter-observer agreement for histogram feature extraction, thereby evaluating measurement repeatability. ICC values were interpreted as poor (<0.40), moderate to good (0.40–0.75), or excellent (>0.75) (26). Features with excellent ICC (>0.75) were retained for subsequent analysis, and the final dataset was constructed using the average values from the two readers.

**Figure 2:**
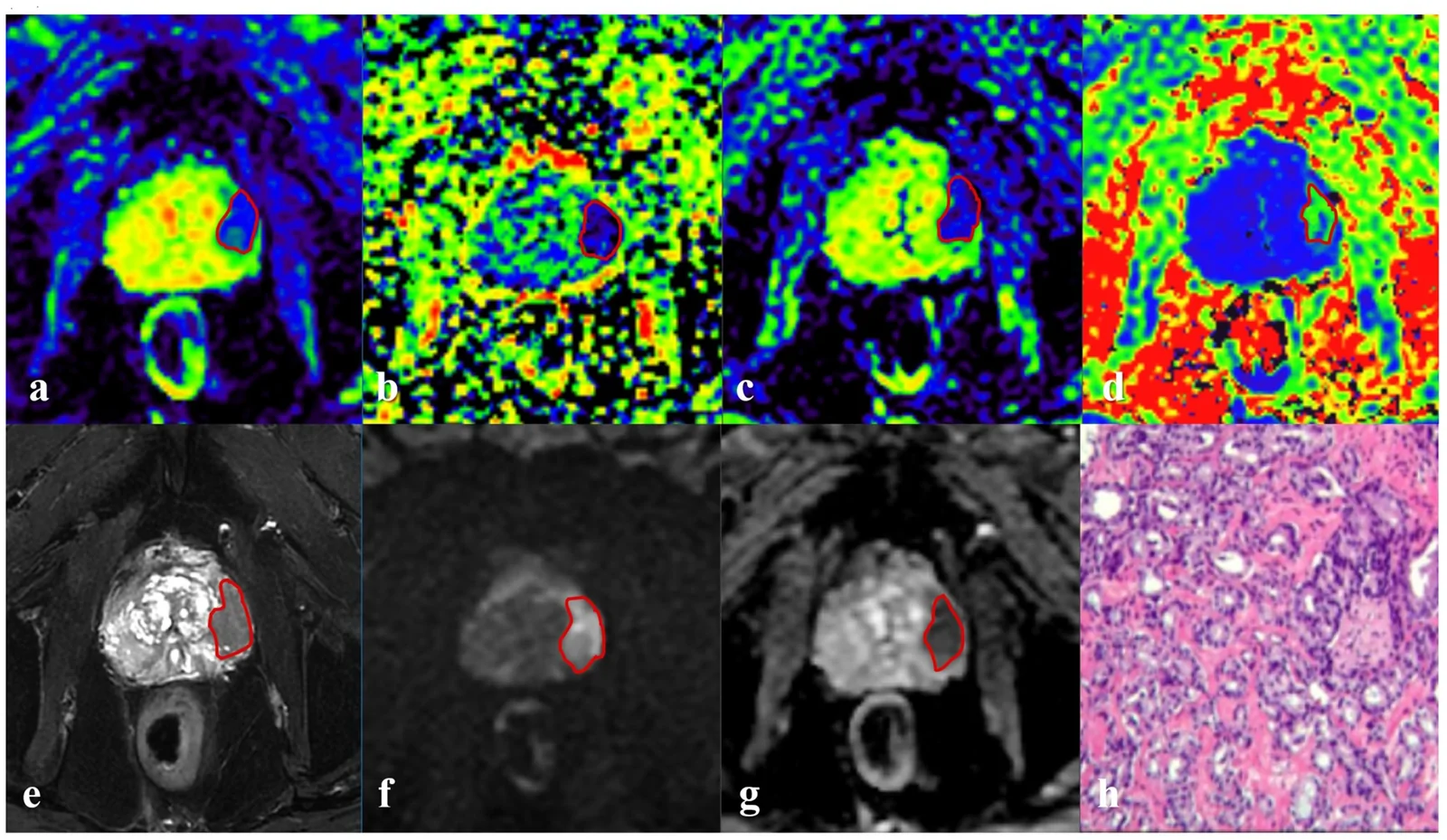
Representative images in a 77-year-old man with ciPCa. **(a–d)** Parametric maps of D, f, MD, and MK. **(e–g)** T2WI, DWI and ADC images. **(h)** Corresponding histopathology (H&E, ×200) showing ISUP grade group 1, pT2 stage, and negative EPE, SVI, PSM, and PNI.

**Figure 3:**
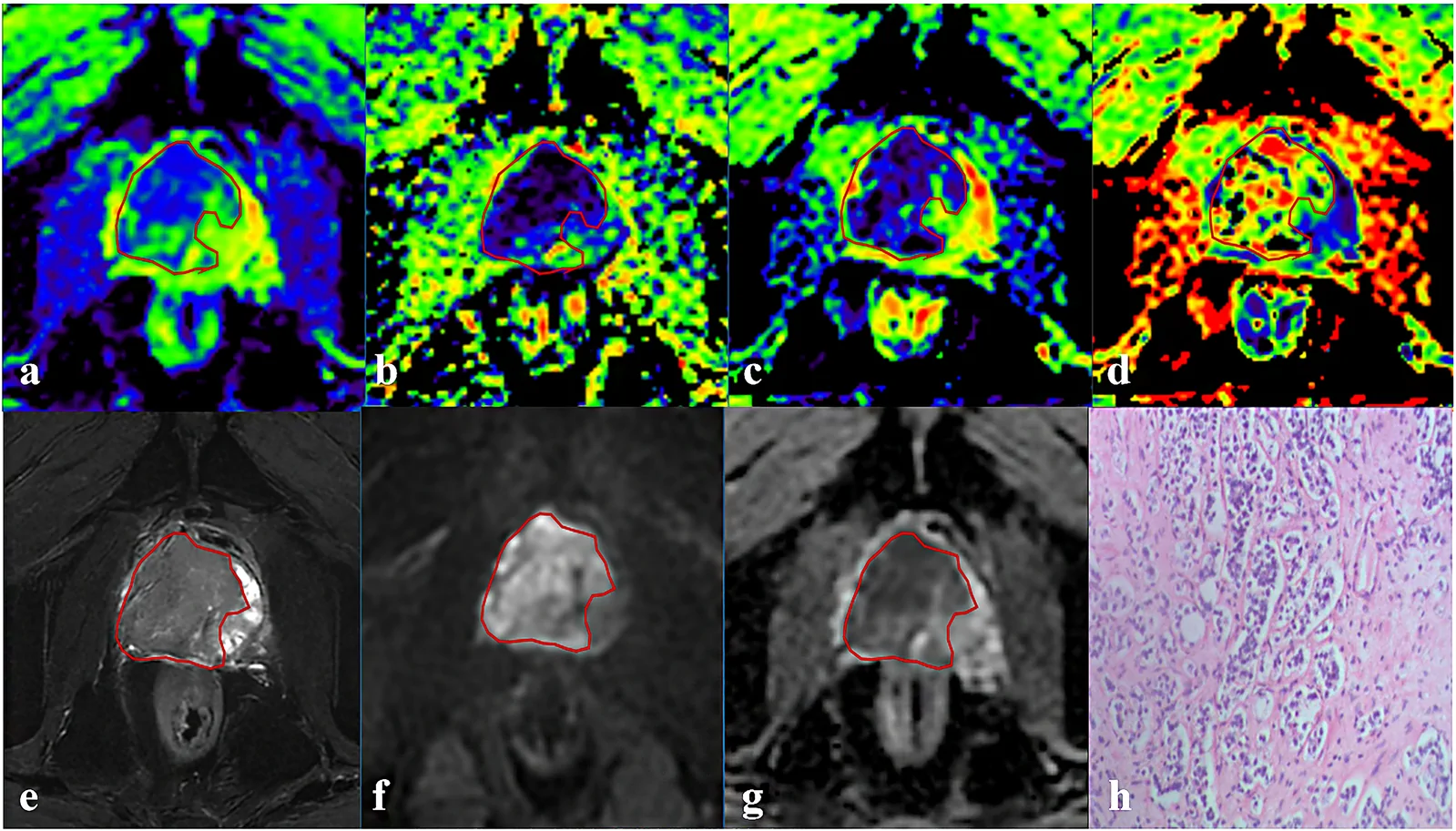
Representative images in a 62-year-old man with csPCa. **(a–d)** Parametric maps of D, f, MD, and MK. **(e–g)** T2WI, DWI, and ADC images. **(h)** Corresponding histopathology (H&E, ×200) showing ISUP grade group 4, pT3b stage, with positive EPE and SVI, and negative PSM and PNI.

### 2.4 Image-histopathological spatial matching and histopathological evaluation

All patients underwent either transrectal ultrasound (TRUS)-guided prostate biopsy or RP. For biopsy cases, a 12-core systematic biopsy scheme was adopted. Under TRUS guidance, one core was obtained from each of six regions per side (base, mid-gland, and apex, both laterally and medially), totaling 12 cores. If suspicious areas were identified on DRE or TRUS, one or two additional cores were obtained. This ensured accurate spatial correspondence between MRI-detected lesions and the sampled biopsy cores. For RP specimens, experienced urogenital pathologists performed whole-mount serial sectioning and lesion localization with standardized slice thickness. MRI-visible lesions were co-registered with whole-mount histological sections using anatomical landmarks and pathological features, including zonal location (peripheral, central, or transition zone), seminal vesicle involvement, anterior prostatic fat pad, and surgical margin distance. Pathologists and radiologists independently and blindly evaluated the spatial correspondence between MRI-detected and whole-mount pathological lesions by visual assessment. For each paired lesion, the following parameters were recorded: ISUP grade group, maximum tumor diameter, tumor volume proportion, EPE, SVI, PNI, and PSM status.

Tissue specimens were fixed in 10% neutral buffered formalin, embedded in paraffin, sectioned, and stained with hematoxylin-eosin (H&E). Immunohistochemical staining was performed according to clinical indications. Postoperative specimens were independently reviewed by two genitourinary pathologists with over 10 years of experience, who were blinded to the imaging findings. The evaluation focused on GS and ISUP GG. Any discrepancies were resolved by consensus. For RP specimens, adverse pathological features—including EPE, SVI, PSM, and PNI—were also evaluated. pT was determined according to the 8th edition of the AJCC TNM staging system (27). These histopathological findings served as the reference standard for imaging analysis.

### 2.5 Statistical analysis

Statistical analyses were performed using SPSS version 26.0 (IBM Corp., Armonk, NY, USA), MedCalc version 20.1 (MedCalc Software Ltd., Ostend, Belgium), and R version 4.5.2 (R Core Team, Vienna, Austria). After Shapiro-Wilk testing, normally distributed variables were expressed as mean ± standard deviation and compared using the independent-samples t-test; non-normally distributed variables were expressed as median (interquartile range [IQR]) and compared using the Mann-Whitney U test. Categorical data were reported as n (%) and compared using the *χ²* or Fisher exact test. Spearman’s rank correlation analysis was performed to assess associations between histogram parameters and both ISUP GG and pT stage. Correlation strength was classified as weak (|*r*| ≤ 0.4), moderate (0.4 < |*r*| ≤ 0.7), or strong (*|r*| > 0.7) (28). Receiver operating characteristic (ROC) curve analysis was used to assess diagnostic performance, with areas under the curve (AUC) and Youden index-maximized cutoffs reported. AUCs were compared using the DeLong test. Combined model development: (a) Using csPCa as the dependent variable, we screened histogram parameters across ADC, DKI, and IVIM sequences using univariable logistic regression, with *P* < 0.05 as the threshold for candidate inclusion. (b) We performed multivariable logistic regression separately for each sequence, using forward stepwise selection based on the likelihood ratio test to identify independent predictors and construct a combined model. (c) We constructed ROC curves and compared AUCs among the combined model, the best single parameter from each sequence, and clinical indicators (PSAD, PSA, and smoking history) using DeLong’s test. (d) We used bias-corrected bootstrap resampling (1,000 replicates) to calculate the optimism-corrected AUC and its 95% confidence interval, thereby assessing model robustness. Statistical significance was set at *P* < 0.05.

## 3 Results

### 3.1 Patient characteristics

This retrospective study enrolled 138 consecutive patients with histopathologically confirmed PCa (78 diagnosed by biopsy only and 60 by RP). Baseline characteristics are summarized in **Table 2**. Smoking history, PSA, and PSAD differed significantly between the two groups (*P* < 0.05), whereas age and body mass index did not (*P* ≥ 0.05). ISUP GG 1–5 were distributed as follows: 30 (21.74%), 20 (14.49%), 26 (18.84%), 53 (38.41%), and 9 (6.52%). Among the 60 radical prostatectomy specimens, pT stage distribution was as follows: pT2 in 16 (26.67%), pT3a in 4 (6.67%), pT3b in 26 (43.33%), and pT4 in 14 (23.33%). EPE, SVI, PSM, and PNI were present in 41 (68.33%), 40 (66.67%), 10 (16.67%), and 13 (21.67%) specimens, respectively.

**Table 2:** Baseline characteristics of the two study groups.

Characteristics	ciPCa (n=30)	csPCa (n=108)	Test statistic	*P value*
age (years)	72 (64, 77)	74 (70, 79)	Z = -1.240	0.215
BMI(kg/m²)	23.36 ± 2.67	22.62 ± 2.60	t = 1.382	0.169
Smoker, n (%)				
ever	11 (36.67)	77 (71.30)	*χ²* = 12.190	<0.001
never	19 (63.33)	31 (28.70)		
PSA(ng/ml)	15.43 (11.08, 28.84)	56.62 (19.59, 100.00)	Z = -4.677	<0.001
PSAD	0.52 (0.29, 0.82)	1.36 (0.55, 2.48)	Z = -3.916	<0.001
PI-RADS score [Cases (%)]				
PI-RADS 1-2	1 (3.33)	0 (0.00)	Z = -6.887	<0.001
PI-RADS 3	11 (36.67)	4 (3.70)		
PI-RADS 4	16 (53.33)	27 (25.00)		
PI-RADS 5	2 (6.67)	77 (71.30)		
Pathological results(n=138)						
ISUP GG1	30 (21.74)	0 (0.00)	Z = -8.704	<0.001
ISUP GG2	0 (0.00)	20 (14.49)		
ISUP GG3	0 (0.00)	26 (18.84)		
ISUP GG4	0 (0.00)	53 (38.41)		
ISUP GG5	0 (0.00)	9 (6.52)		
Specimen types				
Biopsy	18 (60.00)	60 (55.56)	Z = -0.433	0.665
Prostatectomy	12 (40.00)	48 (44.44)		
pT(n=60)				
pT2	16 (26.67)		Z = -8.272	<0.001
pT3a	4 (6.67)			
pT3b	26 (43.33)			
pT4	14 (23.33)			
Extraprostatic extension (n =60)	41 (68.33)			
Seminal vesicles invasion (n=60)	40 (66.67)			
Positive surgical margin (n=60)	10 (16.67)			
Perineural invasion (n=60)	13 (21.67)			

Data are presented as median [IQR], Mean ± SD, or n (%). BMI, body mass index.

### 3.2 Interobserver consistency and pathology subgroup analysis

All histogram parameters demonstrated excellent interobserver consistency. The ICC values (95% CI) were as follows: ADC, 0.936 (0.896–0.961); D, 0.909 (0.854–0.944); f, 0.865 (0.784–0.922); D*, 0.802 (0.727–0.876); MD, 0.916 (0.830–0.957); and MK, 0.870 (0.812–0.901). The subgroup analysis included 42 patients with paired preoperative biopsy and RP specimens. When RP pathology was used as the reference standard, the biopsy ISUP grade group agreed with the RP grade in 38 of 42 patients (90.5%). The linear-weighted Cohen’s κ was 0.940 (95% CI: 0.884–0.997; *P* < 0.001).

### 3.3 Discrimination between csPCa and ciPCa

As shown in **Table 3**, histogram parameters were compared between csPCa and ciPCa. Compared with ciPCa, csPCa exhibited significantly lower values for ADC (mean, median, RMS, minimum, 10th and 90th percentiles), MD (mean, median, 10th percentile, minimum), D (mean, median, RMS, 90th percentile, entropy, IQR, MAD), and f (mean, maximum, RMS, 90th percentile). Conversely, MD_skewness and MK (90th percentile, maximum, minimum, range, RMS) were significantly higher (all *p* < 0.05). No significant differences were observed for D* parameters.

**Table 3:** Comparison of representative histogram parameters between ciPCa and csPCa.

Parameters	ciPCa (n=30)	csPCa (n=108)	Z/t value	*P* value
ADC (×10^-3^mm^2^/s)				
10th percentile	0.690 (0.575, 0.870)	0.587 (0.523, 0.698)	-3.115	0.002
90th percentile	1.092 (0.819, 1.356)	0.905 (0.797, 1.061)	-2.565	0.010
Mean	0.898 ± 0.213	0.760 ± 0.151	3.320	0.002
Median	0.829 (0.678, 1.083)	0.714 (0.646, 0.851)	-2.787	0.005
Minimum	0.500 (0.373, 0.695)	0.393 (0.196, 0.541)	-2.502	0.012
RMS	0.914 ± 0.217	0.774 ± 0.154	3.300	0.002
MD (×10^-3^mm^2^/s)				
10th percentile	0.084 (0.070, 0.102)	0.084 (0.070, 0.102)	-2.793	0.005
Mean	0.122 (0.098, 0.135)	0.104 (0.089, 0.118)	-2.617	0.009
Median	0.119 (0.098, 0.133)	0.103 (0.087, 0.116)	-2.767	0.006
Minimum	0.047 (0.034, 0.070)	0.032 (0.009, 0.047)	-3.428	0.001
Skewness	0.120 (-0.240, 0.450)	0.395 (0.161, 0.750)	-2.826	0.005
MK				
90th percentile	0.122 (0.103, 0.146)	0.138 (0.125, 0.156)	-2.715	0.007
Maximum	0.178 (0.126, 0.222)	0.207 (0.172, 0.295)	-2.924	0.003
Minimum	-0.010 (-0.026, 0.013)	-0.025 (-0.041, 0.010)	-3.020	0.003
Range	0.170 (0.115, 0.234)	0.233 (0.183, 0.324)	-3.252	0.001
RMS	0.090 (0.079, 0.114)	0.108 (0.100, 0.120)	-2.493	0.013
D (×10-3mm2/s)				
90th percentile	1.025 (0.827, 1.266)	0.772 (0.692, 0.903)	-4.279	<0.001
Entropy	4.395 (3.670, 5.095)	3.935 (3.380, 4.478)	-2.578	0.010
IQR	0.169 (0.117, 0.251)	0.121 (0.094, 0.178)	-2.756	0.006
MAD	0.107 (0.079, 0.155)	0.076 (0.057, 0.118)	-2.955	0.003
Mean	0.822 (0.643, 0.920)	0.643 (0.574, 0.737)	-3.675	<0.001
Median	0.814 (0.626, 0.912)	0.640 (0.565, 0.730)	-3.567	<0.001
RMS	0.862 (0.662, 0.928)	0.659 (0.579, 0.756)	-3.944	<0.001
f				
90th percentile	0.281 (0.247, 0.354)	0.232 (0.196, 0.269)	-3.089	0.002
Mean	0.185 (0.133, 0.236)	0.145 (0.115, 0.185)	-2.697	0.007
Maximum	0.523 (0.399, 0.637)	0.407 (0.291, 0.538)	-2.457	0.014
RMS	0.208 (0.175, 0.249)	0.162(0.136, 0.200)	-3.074	0.002

Data are presented as median [IQR] or Mean ± SD. mean absolute deviation. MAD, mean absolute deviation.

### 3.4 Performance comparison of single-parameter and combined-parameter models with clinical indicators

ROC analysis showed that D_90th percentile achieved the highest AUC for differentiating csPCa from ciPCa (AUC = 0.756), outperforming the best-performing features from the other sequences, including ADC_10th percentile (AUC = 0.686) and MD_minimum (AUC = 0.702). Details are provided in **Figure 4a** and **Supplementary Table 1**.

**Figure 4:**
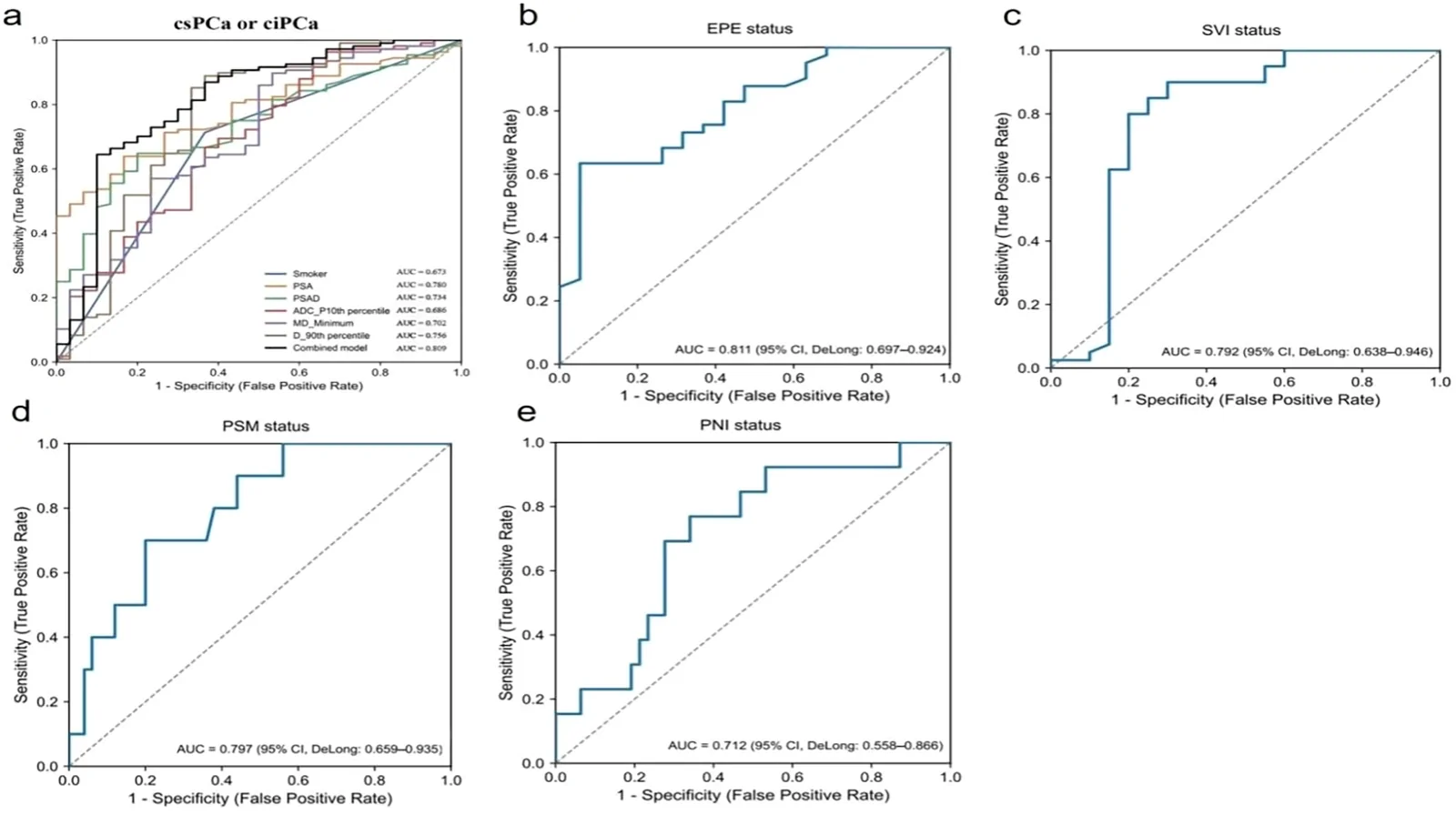
ROC curves for discriminating prostate cancer features. **(a)** Clinical, optimal single-parameter, and combined imaging models for discriminating csPCa from ciPCa. **(b)** EPE status. **(c)** SVI status. **(d)** PSM status. **(e)** PNI status. ROC, receiver operating characteristic.

Based on univariable and multivariable logistic regression analyses (**Tables 4a**, **b**), the following parameters were identified as independent predictors of csPCa from each sequence: ADC_mean, MD_minimum, D_90th percentile, and f_maximum. These parameters were used to construct a combined model. The combined model achieved an AUC of 0.809 (95% CI: 0.712–0.907) for diagnosing csPCa. The DeLong test showed that the combined model significantly outperformed the best single parameter from each sequence as well as traditional clinical indicators (smoking history, PSA, and PSAD) (**Table 4c**; **Figure 4a**). Internal validation using bootstrap resampling (1,000 repetitions) yielded an optimism-corrected AUC of 0.808 (95% CI: 0.697–0.897), indicating good model robustness.

**Table 4a:** Univariate logistic regression analysis (*P* < 0.05).

Parameter	β coefficient	SE	Wald value	*P* value	OR (95% CI)
DWI					
ADC_10th percentile	-0.005	0.001	12.533	<0.001	0.995 (0.992–0.998)
ADC_90th percentile	-0.003	0.001	10.187	0.001	0.997 (0.995–0.999)
ADC_mean	-0.005	0.001	12.858	<0.001	0.995 (0.993–0.998)
ADC_median	−0.004	0.001	11.974	<0.001	0.995 (0.992–0.998)
ADC_minimum	−0.003	0.001	6.976	0.008	0.997 (0.996–0.999)
ADC_RMS	−0.004	0.001	12.696	0.002	0.996 (0.993–0.998)
DKI					
MD_10th percentile	−0.032	0.011	7.948	0.005	0.969 (0.947–0.990)
MD_mean	−0.023	0.009	6.804	0.009	0.977 (0.946–0.994)
MD_median	−0.027	0.009	8.288	0.008	0.973 (0.955–0.991)
MD_minimum	-0.030	0.009	10.600	0.001	0.971 (0.953–0.988)
MD_skewness	1.481	0.511	8.383	0.004	4.397 (1.614–11.981)
MK_90th percentile	0.018	0.0085	4.711	0.030	1.019 (1.002–1.036)
MK_minimum	-0.016	0.006	6.568	0.010	0.984 (0.972–0.997)
IVIM					
D_90th percentile	-0.005	0.001	19.296	<0.001	0.995 (0.992–0.997)
D_entropy	-0.699	0.255	7.486	0.006	0.497 (0.196–0.791)
D_IQR	0.007	0.002	7.564	0.006	0.993 (0.989–0.998)
D_MAD	-0.013	0.004	8.983	0.003	0.987 (0.979–0.996)
D_mean	-0.005	0.001	14.589	<0.001	0.995 (0.992–0.997)
D_median	-0.005	0.001	14.031	<0.001	0.995 (0.992–0.998)
D_RMS	-0.006	0.001	16.352	0.0001	0.994 (0.992–0.997)
f_maximum	-0.003	0.001	6.341	0.012	0.997 (0.995–0.999)

Candidate parameters were screened using univariable logistic regression, with *P* < 0.05 as the significance threshold.

**Table 4b:** Multivariable logistic regression analysis.

Sequence	Parameter	β coefficient	SE	Wald value	*P* value	OR (95% CI)
DWI	ADC_Mean	-0.005	0.001	12.858	0.001	0.996 (0.993–0.998)
DKI	MD_Minimum	-0.027	0.009	9.084	0.003	0.973 (0.956–0.990)
IVIM	D_90th percentile	-0.005	0.001	17.762	<0.001	0.995 (0.992–0.997)
IVIM	f_Maximum	-0.003	0.001	4.561	0.033	0.997 (0.994-0.999)

Forward stepwise selection based on the likelihood ratio test was used to identify the best imaging indicator from each sequence.

**Table 4c:** ROC distribution of the clinical and imaging combined model.

Model	AUC (95%CI)	*P* value	Cut-off value	Sensitivity (%)	Specificity (%)
Smoker	0.673 (0.588–0.751)	0.005	>0.00	71.30	63.33
PSA	0.780 (0.701–0.846)	<0.001	>29.61	63.89	83.33
PSAD	0.734 (0.652–0.806)	<0.001	>0.83	64.81	80.00
Combined model	0.809 (0.712–0.907)	<0.001	>0.84	64.49	90.00

The combined model comprised ADC_mean, MD_Minimum, D_90th percentile, and f_Maximum.

### 3.5 Correlations of histogram parameters with ISUP GG and pT stage

In the entire cohort (n = 138), multiple histogram parameters correlated significantly with ISUP GG (**Figure 5a**). Specifically, MK (90th percentile, minimum, maximum, and RMS), along with MD_skewness, showed positive correlations, whereas all remaining parameters were negatively correlated (all *p* < 0.05). D_RMS exhibited the strongest correlation (*r* = −0.458, *p* < 0.001). **Figure 6a** illustrates D_RMS distributions across ISUP grade groups.

**Figure 5:**
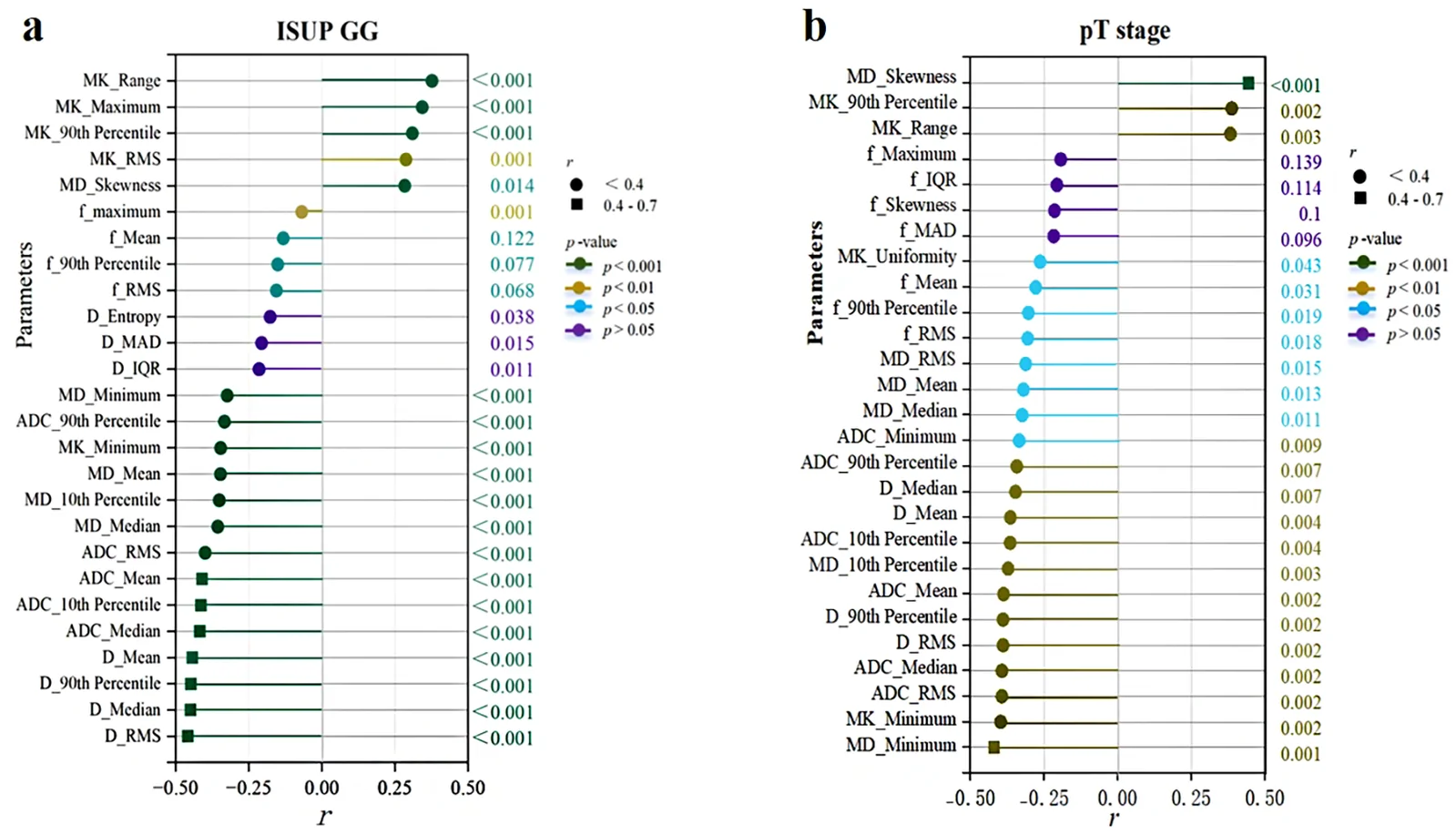
Correlations between multi-parametric MRI histogram parameters and **(a)** ISUP GG and **(b)** pT stage in PCa. MAD, mean absolute deviation.

**Figure 6:**
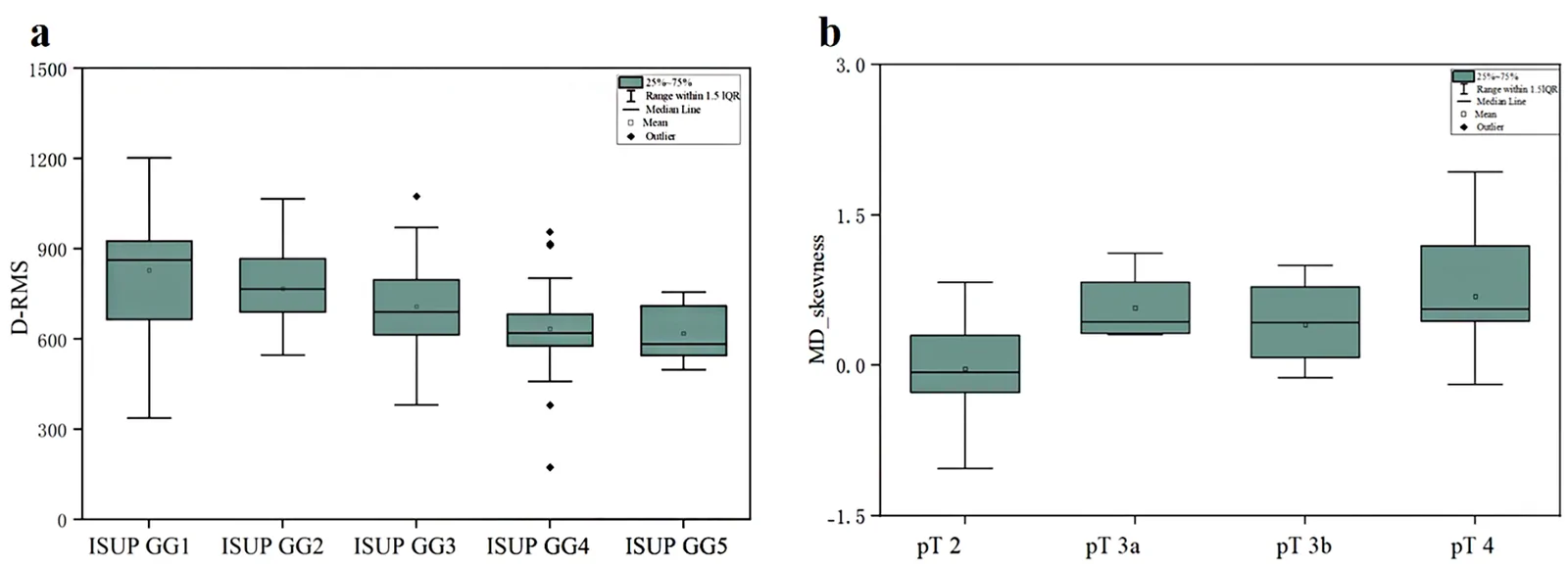
Box plots illustrating the distributions of D_RMS and MD_skewness across pathological grade and stage. **(a)** Comparison of D_RMS values among different ISUP grade groups. **(b)** Comparison of MD_skewness values among different pT stages.

In the surgical cohort (n = 60), correlations between histogram parameters and pT stage were further assessed (**Figure 5b**). MK(90th percentile and range) and MD_skewness were positively correlated with pT stage, whereas all other parameters were negatively correlated (all *p* < 0.05). MD_skewness exhibited the strongest correlation (*r* = 0.443, *p* < 0.001). **Figure 6b** illustrates MD_skewness distributions across pT stages.

### 3.6 Histogram analysis for differentiating EPE, SVI, and PSM, PNI

The ability of multi-parametric histogram features to differentiate EPE, SVI, PSM, and PNI was assessed in 60 surgical patients.

Compared with the EPE-negative group, the EPE-positive group showed significantly lower ADC and MD (mean, median, RMS, 10th/90th percentiles, minimum), D (mean, median, RMS), and f (mean, RMS, 90th percentile) (all *P* < 0.05), whereas ADC_skewness, MD (kurtosis, skewness) and MK_range were significantly higher (all *P* < 0.05). MD_skewness yielded the highest AUC for EPE (0.811; **Figure 4b**).

For SVI, the positive group exhibited significantly lower ADC and MD. Additionally, D(mean, median, RMS, 10th and 90th percentiles) and f (mean, RMS, 90th percentile) were significantly lower in the positive group (all *P* < 0.05), whereas MD(skewness, range) and MK(mean, median, RMS, 90th percentile, maximum, uniformity, range) were significantly higher (all *P* < 0.05). MK_90th percentile yielded the highest AUC for SVI (0.792; **Figure 4c**).

For PSM, the positive group showed significantly lower ADC (10th percentile, minimum), MD (mean, RMS, 90th percentile), and D_10th percentile (all *P* < 0.05), whereas MD_kurtosis was significantly higher (all *P* < 0.05). ADC_10th percentile yielded the highest AUC for PSM (0.797; **Figure 4d**).

For PNI, the positive group showed significantly lower f (mean, median, RMS, 90th percentile, IQR) (*P* < 0.05), and f_RMS yielded the highest AUC for PNI (0.712; **Figure 4e**).

## 4 Discussion

This study evaluated the clinical value of histogram features derived from 5.0-T multi-parametric diffusion MRI for assessing ISUP GG and adverse pathological features in PCa. ADC_mean, MD_minimum, D_90th percentile, and f_maximum were identified as the best independent predictors for differentiating ciPCa from csPCa in their respective sequences. The combined model incorporating these four parameters significantly outperformed any single parameter. Multiple histogram parameters derived from ADC, MD, MK, D, and f maps showed mild-to-moderate correlations with ISUP GG and pT stage, with D_RMS showing the strongest correlation with ISUP GG and MD_skewness with pT stage. For adverse pathological factors, several histogram parameters reflecting central tendency and dispersion differed significantly between patients with and without EPE, SVI, PSM, and PNI. Specifically, MD_skewness, MK_90th percentile, f_RMS, and ADC_10th percentile achieved the highest AUC values for EPE, SVI, PSM, and PNI, respectively. These findings suggest that histogram features derived from 5.0-T mpMRI hold promise for the noninvasive preoperative assessment of PCa aggressiveness and adverse prognostic factors.

Quantitative histogram analysis reduces reliance on subjective morphological criteria in PI-RADS reporting, enabling objective quantification of tumor heterogeneity and invasiveness. In this study, most histogram features differed significantly between csPCa and ciPCa. Among single parameters, D_90th percentile had the highest AUC (0.756). This is consistent with Das et al. (29); additionally, evidence indicates that higher percentiles are more sensitive in capturing invasive features at tumor margins and extreme diffusion values in the core, reflecting greater sensitivity to intratumoral skewed distributions (30). Multivariable logistic regression identified ADC_mean, MD_minimum, D_90th percentile, and f_maximum as independent predictors of csPCa derived from DWI, DKI, and IVIM sequences. ADC_mean reflects average water diffusion restriction within lesions; higher Gleason scores correlate with denser cell packing and an elevated nuclear-to-cytoplasmic ratio, restricting Brownian motion (31). MD_Minimum reflects the most severe diffusion restriction within lesions, supporting its role as a marker of cellular density and malignancy (32). D_90th percentile captures extreme IVIM pure diffusion coefficient values, revealing invasive features at the tumor core and periphery (30). The perfusion fraction f reflects the proportion of microcirculatory perfusion relative to total diffusion signal, indicating microvascular proliferation; f_Maximum, as the upper limit, reflects vascular complexity and density of csPCa (33). In summary, these four parameters characterize csPCa across complementary microscopic dimensions, including single-exponential diffusion, lower-limit non-Gaussian diffusion, diffusion heterogeneity, and upper-limit perfusion.

Further correlation analysis showed mild-to-moderate correlations between multiparametric histogram features and ISUP GG and pT stage. Features reflecting microstructural complexity and heterogeneity—specifically MK parameters (central tendency and dispersion) and MD dispersion parameters—were positively correlated with grade and stage. These higher values indicate greater complexity and invasiveness (34). Conversely, features reflecting restricted water diffusion (overall distribution of ADC, MD, and D) were negatively correlated with grade and stage. This indicates greater cell density and more pronounced diffusion restriction in higher-grade/stage tumors (35). Furthermore, f distribution features reflecting microvascular perfusion also correlated negatively. This may reflect ineffective perfusion due to aberrant angiogenesis in higher-grade/stage tumors, characterized by vascular disorganization and impaired perfusion efficiency (36).

Current identification of adverse pathological factors (EPE, SVI, PSM, and PNI) relies primarily on subjective morphological assessment (e.g., capsular irregularity, seminal vesicle signal abnormalities, direct tumor invasion, and neurovascular bundle asymmetry) and signal-based observations. Quantitative mp-dMRI may provide useful imaging data that could aid in evaluating these pathological features. Previous studies (28, 37, 38) have demonstrated that mp-dMRI may help assess tumor aggressiveness and heterogeneity.

In this study, central tendency metrics (mean, median, RMS) of ADC, MD, D, and f were significantly lower in EPE- and SVI-positive groups, suggesting that Gaussian (DWI), non-Gaussian (DKI), and IVIM biexponential models can all assess invasive PCa behavior by quantifying water diffusion restriction. IVIM additionally provides complementary microcirculatory information. Increased cell density and microstructural disorganization lead to more pronounced diffusion restriction and microcirculatory impairment, reflected by expansion of low-value regions, leftward histogram shift, and reduced high-value proportions. Lower histogram parameters thus indicate poorer diffusion and perfusion, correlating with more severe adverse features (39). Consistent with our results, Chen et al. (28) reported lower ADC mean and median in patients with EPE or SVI. Furthermore, in EPE- and SVI-positive groups, ADC and MD dispersion metrics (kurtosis, skewness) increased; in the SVI-positive group, MK central tendency (mean, median, RMS) and dispersion metrics (percentiles, range, uniformity) were also elevated, indicating increased microstructural complexity and heterogeneity in invaded tissue (40). Specifically, MD_skewness and MK_90th percentile achieved the highest AUCs for EPE and SVI, respectively.

Additionally, MD central tendency metrics and dispersion parameters of ADC, MD, and D (ADC_10th percentile, MD_90th percentile, D_10th percentile) were reduced in the PSM-positive group, consistent with the aforementioned distribution characteristics. Meng et al. (37) also reported reduced MD in the PSM-positive group, consistent with our findings. However, f values did not differ significantly between PSM-positive and PSM-negative groups. We hypothesize that preexisting hypoperfusion in PSM-positive regions may reduce the sensitivity of f to perfusion changes; Notably, ADC_10th percentile achieved the highest AUC for identifying PSM.

By contrast, in the PNI-positive group, only f central tendency metrics (mean, median, and RMS) decreased. ADC, MD, MK, and D showed no significant between-group differences, suggesting limited sensitivity of these diffusion parameters to microstructural changes in invaded neurovascular bundles (41). Only the perfusion fraction detected microcirculatory changes following neurovascular bundle invasion, and f_RMS achieved the highest AUC. In summary, multidimensional information from DWI, DKI, and IVIM—reflecting cellular density, microstructural complexity, and perfusion/diffusion—may provide complementary value in identifying adverse pathological features of PCa. This integrated approach may allow comprehensive characterization of heterogeneity across different pathological factors and aid in their differentiation.

This study presents three main innovations. First, we applied 5.0-T ultra-high-field MRI to assess PCa grading, pathological staging, and adverse prognostic factors. Previous studies (21, 22) have shown that, compared with 3.0-T MRI, 5.0-T MRI provides higher signal-to-noise ratio and spatial resolution, allowing clearer visualization of microstructural details at tumor margins and the interfaces between these margins and adjacent structures (e.g., prostatic capsule, seminal vesicle walls, and periprostatic nerve tracts). This improved image quality may potentially benefit the reliability of quantitative measurements. Second, we integrated three diffusion models—DWI, DKI, and IVIM—to systematically address the limitations of single-model approaches in characterizing tumor heterogeneity. These models capture distinct physiological aspects of the tumor microenvironment. Together, these complementary approaches establish a multidimensional quantitative framework that simultaneously characterizes tumor perfusion, cellularity, and structural complexity, providing a comprehensive, reliable, noninvasive preoperative assessment of PCa aggressiveness (42). Third, we preliminarily explored the utility of MRI combined with histogram parameters in evaluating adverse pathological factors in PCa. Our initial findings suggest that this approach holds promise as a source of novel insights and supportive evidence for the noninvasive assessment of adverse prognostic factors in PCa.

This study has several limitations. First, its single-center, retrospective design may introduce selection bias, and the sample size of surgical patients was relatively limited, with particularly few positive cases of PSM and PNI. Exploring “optimal” histogram features within these small subgroups may yield unstable estimates. Future multicenter, large-sample prospective studies are warranted to validate these findings. Second, pathological diagnosis in some patients relied on needle biopsy, which is susceptible to sampling errors that may bias GS and ISUP GG assignments and thus compromise diagnostic accuracy. Future studies could improve reliability by using postoperative pathology as the reference standard or by performing sensitivity analyses. Third, we did not perform a head-to-head comparison between 5.0-T and lower-field-strength MRI (e.g., 3.0-T or 1.5-T). Consequently, we cannot claim that the observed clinical benefits are attributable to the higher field strength. Future prospective comparative studies are needed to determine the incremental diagnostic value of 5.0-T MRI over that of conventional field strengths. Fourth, our assessment of tumor heterogeneity relied exclusively on first-order histogram features, which characterize the distribution of voxel intensities. Unlike higher-order texture features, these metrics do not capture the spatial relationships between voxels or the underlying structural information of the tumor. Future large-scale studies should integrate first-order histogram features with higher-order texture analysis to construct more comprehensive radiomics models.

## 5 Conclusion

Quantitative histogram analysis using 5.0-T ultra-high-field mp-dMRI effectively characterizes tumor heterogeneity and aggressiveness in PCa and demonstrates correlations with ISUP GG and pT stage. Furthermore, preliminary results suggest that this technique may be useful for identifying adverse pathological features, including EPE, SVI, PSM, and PNI. This approach provides added value to preoperative PCa diagnosis and the assessment of adverse prognostic factors.

## Data Availability

The original contributions presented in the study are included in the article/**Supplementary Material**. Further inquiries can be directed to the corresponding authors.
