# Supplementary material for: 5.0T multi-parametric diffusion MRI histogram analysis for prostate cancer pathology grading and adverse pathological factors
Source: Front Oncol. 2026 Sep 29;16:1957407. doi: 10.3389/fonc.2026.1957407 (PMC13657985; doi:10.3389/fonc.2026.1957407)
Supplement: Supplementary file 1 [file Table1.docx]

Supplementary Table 1. Diagnostic performance of ADC, MD, MK, D, and f parameters for prostate cancer.

| Parameter | AUC (95%CI) | *P* value | Cut-off value | Sensitivity (%) | Specificity(%) | Youden Index |
| --- | --- | --- | --- | --- | --- | --- |
| ADC |  |  |  |  |  |  |
| 10th percentile | 0.686 (0.602-0.763) | 0.001 | ≤629.00 | 66.67 | 63.33 | 0.300 |
| 90th percentile | 0.653 (0.568-0.732) | 0.015 | ≤1180.00 | 89.81 | 46.67 | 0.365 |
| Mean | 0.673 (0.588-0.750) | 0.005 | ≤963.68 | 92.59 | 43.33 | 0.359 |
| Median | 0.667 (0.581-0.745) | 0.006 | ≤967.00 | 94.44 | 40.00 | 0.344 |
| Minimum | 0.650 (0.564-0.729) | 0.012 | ≤413.00 | 53.7 | 73.33 | 0.270 |
| RMS | 0.669 (0.583-0.746) | 0.007 | ≤974.19 | 92.59 | 43.33 | 0.359 |
| MD |  |  |  |  |  |  |
| 10th percentile | 0.664 (0.579-0.743) | 0.007 | ≤79.28 | 70.09 | 60.00 | 0.301 |
| Mean | 0.662 (0.577-0.741) | 0.006 | ≤122.69 | 80.37 | 53.33 | 0.337 |
| Median | 0.670 (0.585-0.748) | 0.004 | ≤110.88 | 68.22 | 66.67 | 0.349 |
| Minimum | 0.702 (0.618-0.777) | <0.001 | ≤55.07 | 89.72 | 46.67 | 0.364 |
| Skewness | 0.671 (0.586-0.748) | 0.007 | >-0.13 | 92.59 | 40.00 | 0.326 |
| MK |  |  |  |  |  |  |
| 90th percentile | 0.662 (0.577-0.740) | 0.019 | >107.36 | 93.52 | 43.33 | 0.369 |
| Maximum | 0.675 (0.590-0.752) | 0.004 | >150.62 | 90.74 | 46.67 | 0.374 |
| Minimum | 0.681(0.596-0.757) | 0.001 | ≤-14.23 | 73.15 | 60.00 | 0.332 |
| Range | 0.694 (0.610-0.770) | 0.001 | >132.89 | 95.37 | 36.67 | 0.320 |
| RMS | 0.649 (0.563-0.728) | 0.024 | >90.15 | 84.26 | 53.33 | 0.376 |
| D |  |  |  |  |  |  |
| 90th percentile | 0.756 (0.675–0.825) | <0.001 | ≤1000.41 | 88.89 | 63.33 | 0.522 |
| Entropy | 0.654 (0.569–0.733) | 0.013 | ≤4.74 | 87.04 | 40.00 | 0.270 |
| IQR | 0.677 (0.592–0.754) | 0.002 | ≤74.70 | 50.00 | 83.33 | 0.333 |
| MAD | 0.665 (0.580–0.743) | 0.005 | ≤212.00 | 87.96 | 40.00 | 0.280 |
| Mean | 0.720 (0.637–0.793) | <0.001 | ≤784.05 | 81.48 | 60.00 | 0.415 |
| Median | 0.713 (0.630–0.787) | <0.001 | ≤791.25 | 86.11 | 56.67 | 0.428 |
| RMS | 0.736 (0.654–0.807) | <0.001 | ≤804.83 | 84.26 | 63.33 | 0.476 |
| f |  |  |  |  |  |  |
| 90th percentile | 0.685 (0.600–0.761) | 0.001 | ≤262.85 | 72.22 | 73.33 | 0.456 |
| Mean | 0.661 (0.576–0.740) | 0.007 | ≤167.27 | 68.52 | 70.00 | 0.385 |
| Maximum | 0.647 (0.561–0.726) | 0.009 | ≤395.95 | 48.15 | 80.00 | 0.282 |
| RMS | 0.684 (0.599–0.760) | 0.002 | ≤184.19 | 69.44 | 76.67 | 0.461 |

Note: Cut-off values are the optimal thresholds determined by the Youden index.
